# Improving Object Tracking by Added Noise and Channel Attention

**DOI:** 10.3390/s20133780

**Published:** 2020-07-06

**Authors:** Mustansar Fiaz, Arif Mahmood, Ki Yeol Baek, Sehar Shahzad Farooq, Soon Ki Jung

**Affiliations:** 1 School of Computer Science and Engineering, Kyungpook National University, Daegu 41566, Korea; mustansar@knu.ac.kr (M.F.); qkkndq@knu.ac.kr (K.Y.B.); sehar146@knu.ac.kr (S.S.F.); 2 Department of Computer Science, Information Technology University, Lahore 54000, Pakistan; arif.mahmood@itu.edu.pk

**Keywords:** Siamese networks, convolutional neural network, visual tracking, noise regularization, attentional mechanism

## Abstract

CNN-based trackers, especially those based on Siamese networks, have recently attracted considerable attention because of their relatively good performance and low computational cost. For many Siamese trackers, learning a generic object model from a large-scale dataset is still a challenging task. In the current study, we introduce input noise as regularization in the training data to improve generalization of the learned model. We propose an Input-Regularized Channel Attentional Siamese (IRCA-Siam) tracker which exhibits improved generalization compared to the current state-of-the-art trackers. In particular, we exploit offline learning by introducing additive noise for input data augmentation to mitigate the overfitting problem. We propose feature fusion from noisy and clean input channels which improves the target localization. Channel attention integrated with our framework helps finding more useful target features resulting in further performance improvement. Our proposed IRCA-Siam enhances the discrimination of the tracker/background and improves fault tolerance and generalization. An extensive experimental evaluation on six benchmark datasets including OTB2013, OTB2015, TC128, UAV123, VOT2016 and VOT2017 demonstrate superior performance of the proposed IRCA-Siam tracker compared to the 30 existing state-of-the-art trackers.

## 1. Introduction

Visual Object Tracking (VOT) is a promising and fundamental research area in computer vision applications including robotics [1], video understanding [2], video surveillance [3] and autonomous driving [4]. Given the initial state of a target object (generally specified by a bounding box) in a video, the aim of an object tracker is to estimate the spatial trajectory of the target object in the upcoming frames. Despite a significant progress made in the field of VOT, it remains a challenging problem owing to diverse real-world challenges such as scale variations, occlusion, background clutter, fast motion, and illumination variations.

Deep trackers take the benefits from pretrained deep neural networks and have shown outstanding performance [5,6,7,8,9,10]. These deep trackers extract features from off-the-shelf pretrained models as a backbone feature extractor known as deep features for better discrimination. The pretrained models are trained over ImageNet [11] for image classification tasks such as VGGNet and AlexNet. Many computer vision sub-fields employ pretrained models to benefit from transfer learning [12,13]. However, it can be observed that during tracking, these models may not fully adapt the specific target features and online learning may steer to overfitting [14]. Recently, deep Siamese-based trackers [10,15,16,17] have become popular since they achieve good performance with relatively low computational cost. Deep neural networks are composed of multiple hidden layers, which enable learning complex relationships between the inputs and outputs. However, due to limited training data, deep network models are prone to over-learn the training dataset which may lead to overfitting problem [18]. Dropout [18] and additive noise [19] can be employed to handle this issue in deep neural networks. There exists many approaches to handle overfitting problem by using spatio-residual modules [20], regularizers [5,21], integrating context information [22], or factorized convolution [23]. Another limitation is that online learning is an expensive process and requires more computational resources.

Our proposed methodology tackles aforementioned challenges by adopting an input regularization using similarity matching learning function. To validate the basic concept, we used SiameseFC [15] as our baseline tracker. It is important to improve the training process by including novel data augmentation techniques to enhance the generalization ability of deep trackers. We propose data augmentation by introducing noise into the training dataset. Introducing noise is similar to instructing a network not to change its output and it may be considered a special kind of input regularization. The proposed data augmentation method increases the accuracy and reduces the generalization error and overfitting problem.

Certain convolutional feature channels contribute more than the others, employing a channel attention mechanism can enhance the tracking performance. A channel attention mechanism is considered to be a process of weighting specific features because of their potential to model context information. The inclusion of attention mechanism has already been shown to be beneficial in visual object tracking. ACFN [24] used spatial attention to select a subset of correlation filters for tracking. RASNet [25] employs different kinds of attention models to highlight the dominance or weakness of channels. It is necessary to suppress irrelevant channels while providing higher weights to the more useful channels. Based on these observations, we incorporate a channel attentional mechanism within Siamese framework to enhance the tracking performance. We adapted feature fusion and a special kind of attention mechanism in our tracking framework to generate more discriminative target features.

In the current work, we propose an Input-Regularized Channel Attentional Siamese (IRCA-Siam) network to learn efficient target boosted features and enhance its discriminative ability. Early feature fusion is helpful for encoding adaptive target representations while suppressing noisy information. Moreover, the proposed network exploits the relationship among feature channels at a high level to learn informative and meaningful channels while suppressing trifling channels. The proposed tracker is evaluated over OTB2013 [26], OTB2015 [27], temple color-128 [28], UAV123 [29], VOT2016 [30], and VOT2017 [31] datasets and compared with 30 state-of-the-art methods. The proposed tracker has consistently shown improved performance compared to these trackers.

We summarize our main contributions as follows:

- We propose an additive noise as input regularization to improve deep network generalization.
- Early feature fusion mechanism is proposed to learn better target feature representation.
- An adaptive channel attention mechanism is integrated to give more weight to the important channels compared to the less important ones using a skip connection.
- Robustness of the proposed tracker is evaluated on the six benchmark datasets. Our experiments demonstrate better performance of the proposed tracker compared to the 30 state-of-the-art methods.

## 2. Related Work

In this section, we review different deep learning methods using additive noise. We also discuss closely related tracking approaches including deep features based trackers, Siamese-based and attention-based trackers. A detailed study may be found in recent surveys [6,9,32,33].

### 2.1. Deep Learning with Noise

Deep Neural Network (DNN) models have shown significant importance due to improved performance in various computer vision problems such as image classification, semantic segmentation, and action recognition. However, due to limited training data, networks may lead to overfitting. Dropout is an often used method to handle overfitting issue by randomly dropping out values in the hidden units in the network model [18]. However, it is still unclear how to select the best dropout rate to perform well and how can we maximize the benefit from optimization as well as preventing model from overfitting [19]. Instead of using dropout, many researchers used additive noise to handle overfitting problem [19,34,35]. Increased dropout rate may cause information loss especially when target size is small and decreased dropout may not be able to avoid overfitting. Noh et al. [19] used additive noise as regularizer from marginalized noise instead of dropout approach. Bishop et al. [34] showed that additive noise effect is similar to Tikhonove regularization. Liu et al. [36] used noise layer to prevent their network from adversarial attacks. Fiaz et al. [6] studied the performance of trackers on noisy inputs during tracking. In contrast, we propose an additive noise as input regularization to improve the generalization error in the visual object tracking domain. Proposed regularization improves the tracking performance during the inference. We also verified the performance of our framework by inducing a noise layer before each convolutional layer. Experimental results showed that inducing a noise layer for each convolutional layer reduces the tracking performance compared to adding noise in the input data.

### 2.2. Deep Feature-Based Trackers

Recently, deep learning approaches have boosted the tracking performance due to their inherent characteristics. However, employing deep learning in visual tracking have several limitations. For example, deep learning requires more computational resources and have higher time complexity. The ground truth for the reference target object is provided only on the first frame of the video. To benefit from deep learning and limited available training data, deep features are combined into correlation filter tracking to boost the tracking performance. For instance, DeepSRDCF [5], CF2 [8], and FCNT [37] take the leverage from deep learning by extracting deep features at multiple layers from pretrained models such as VGG [38] or AlexNet [39]. Deep features from different layers were exploited to enable the capabilities of accuracy and robustness for the visual tracking [7,23,40,41]. Bhat et al. [41] revealed that pretrained models do not always fetch performance boost due to incompatible resolutions, unseen target objects and increasing dimensions. On the other hand, deep learning can also be used as classification or regression networks for visual tracking [22,42,43]. CNN-SVM [44] employs CNN model and performs classification task using SVM with saliency map. The TSN tracker [45] used CNN to encode temporal and spatial information for classification. The MDNet [21] is a multi-domain online deep tracker performing tracking as classification task, and capturing the domain dependent information during online tracking within a particle filter framework.

The online model update is performed to adapt different appearance variations of the target, but it may lose target under scenarios such as occlusion, deformation, or background clutter. Online learning requires extra computational cost to update the model parameters. Although CNN-based models have fewer parameters than RNN-based models, frequent model update incur extra computational cost therefore, such trackers may have limited real-world applications.

### 2.3. Siamese Network-Based Trackers

A Siamese network comprises of two parallel Convolutional Neural Networks (CNN) streams that are used to learn the similarity between input images in embedded space and to fuse them to produce an output [46]. Owing to their inherent characteristics such as accuracy and speed, Siamese networks are popular in the visual tracking community [10,15,16,17,47]. A SiameseFC [15] extracts input image features using an embedded CNN model and fuses them by using a correlation layer, to generate a response map. CFNet [10] is an improved version of the SiameseFC and it integrates a correlation filter layer as a differentiable layer within template branch. On the other hand, GOTURN [16] involves the use of a Siamese network as a feature extractor and the use of fully connected layers for fusing embedded features. The GOTURN tracker performs regression between two consecutive frames. The SINT [17] formulates the tracking problem as a verification task to learn the similarity between inputs. These approaches have secured much importance due to their performance, but overfitting might occur if trained on small datasets. The proposed tracking algorithm enhances the discriminative ability of Siamese tracking framework by exploring data augmentation using additive noise.

### 2.4. Attention Mechanism-Based Trackers

Recently, attention mechanisms have become popular owing to their improved learning capabilities. CSRDCF [48] constructs a unique spatial reliability map to impose constraints on correlation filters within a correlation tracking framework. AFS-Siam [49] selects the discriminative kernels from different convolutional layers. Choi et al. [24] proposed ACFN and used spatial attention to select a subset of correlation filters for visual object tracking. RTT [50] used multi-directional recurrent filters to learn the target object appearance. The objective of using a channel attention mechanism has enabled the tracker to learn the most critical information to adapt the target appearance. However, the attention mechanism within convolutional layers has not been fully exploited. On the basis of these considerations, we introduced a channel attention mechanism to highlight the importance of discriminative features. Our technique showed high performance by offline learning efficient discriminative features.

## 3. The Proposed Input-Regularized Channel Attentional Siamese (IRCA-Siam) Network

**Figure 1:**
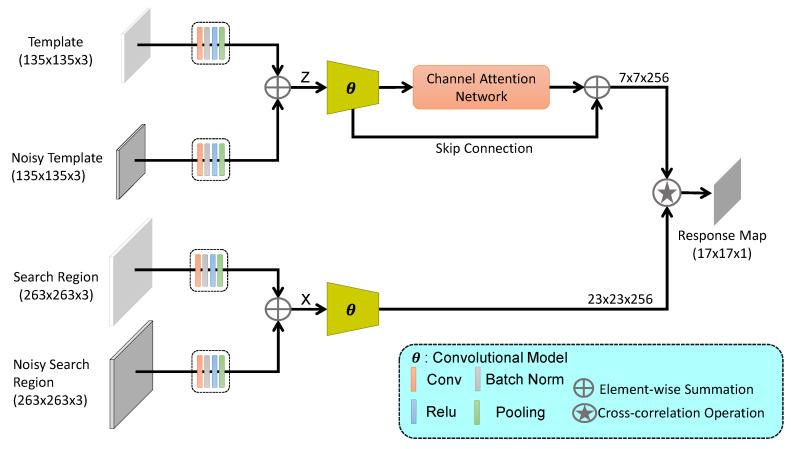
Proposed IRCA-Siam tracking framework. The inputs are fused after MaxPool layer for exemplar and search branches. Channel attentional network is integrated for exemplar branch using a skip connection.

Overall framework of the proposed IRCA-Siam network is shown in Figure 1. Compared to the previous deep trackers, IRCA-Siam exploits additive noise in the input data within Siamese framework to handle overfitting problem. We propose an early feature fusion mechanism for better target localization. We also integrate a channel attention mechanism within IRCA-Siam to highlight the more useful and discriminative features for improved tracking.

### 3.1. Fully Convolutional Siamese Network

The building block of the proposed framework is SiameseFC tracker proposed by Bertinetto et al. [15]. SiameseFC formulates the tracking problem, to learn a similarity map from embedded CNN models, as a cross-correlation problem within a Siamese network architecture. The embedded CNN model consists of two parallel branches, one representing the target and the other representing the search region. In visual tracking, the target template is provided in the first frame of the as an exemplar *z*. The objective of SiameseFC is to find the most similar region from the search region (larger in size than the template) *x* for subsequent frames as:

(1) g(z,x)=θ(z)∗θ(x)+b,

where ∗ represents the cross-correlation, θ(·) denotes the embedded space, and *b* represents the offset of the similarity value. From Equation (1), we note that SiameseFC uses feature representation and discriminative learning to produce a similarity map by using a single function θ(·). The performance of both tasks may lead to overfitting the model to the training data. We therefore propose noisy regularized feature fusion to overcome the challenges faced by SiameseFC and to improve the generalization capability of the tracker. We also highlight the importance of discriminative channel feature information.

### 3.2. Input Regularization and Feature Fusion

In the current study, a data augmentation mechanism is introduced for Siamese networks to overcome their limitations. Existing Siamese trackers suffer due to low fidelity of the target representation. We propose an input regularization during the training of Siamese trackers. Introducing noise into the input can be regarded as input regularization, and it encourages the model to learn various aspects of the object and increases its robustness against noise during testing. The features from both branches are fused (as shown in Figure 1) such that the model can learn the target features under noise or disturbance to enhance its accuracy in real-world noisy environment. It may be noted that during tracking, a target may observe noise leading to performance degradation. The proposed feature fusion mechanism helps to overcome this limitation.

We induce random Gaussian noise into the input patches to obtain noisy images with mean μ and standard deviation σ. A Gaussian noise map $G↪{\mathrm{Rand}}_{G}\left(\mu ,\;\sigma^{2}\right)$ is constructed and added with the input, where ${\mathrm{Rand}}_{G}\left(\cdot \right)$ is a random number generator function based on Gaussian density function. In contrast to existing Siamese networks, the proposed model accepts four inputs, namely a target patch (*z*), a noisy target patch (G+z), a search patch (*x*), and a noisy search patch (G+x). Low-level features from noisy and clean images are fused to encode the spatial target information for better localization.

In practice, we fuse features from target patch and noisy target patch as:

(2) Z=B(z)+B(G+z),

where B represents a convolutional block including a convolutional layer, a normalization layer, a rectifier layer, and a pooling layer. Similarly, features from search and noisy search patches are fused as:

(3) X=B(x)+B(G+x),

The proposed framework is summarized as:

(4) g(z,x)=(Δ(θ(Z))⊕θ(Z))∗θ(X)+b,

where Δ(.) denotes the channel attention and ⊕ represents the element-wise addition operation. The channel attention network is explained in Section 3.3.

During testing, we do not require noisy template and noisy search region. Instead, we provide the same template and search region that are provided to the other two inputs.

### 3.3. Channel Attention Network

A convolutional feature channel can be considered to be equivalent to a specific type of visual pattern. SiameseFC treats the feature channels for both the exemplar and search branches equally, which leads to performance degradation. However, the proposed channel attention mechanism exploits the relationship among channels and assigns more weights to channels that contribute more to target discrimination and localization. The objective is to enhance the adaptation capacity of the model to capture target variations. We incorporate a channel attention mechanism in the template branch as shown in Figure 1. There exists many channel attentional networks to calibrate the channel information such as SENet [51] and SA-Siam [52] which employ only global max-pooling and multi-perceptron layer. Choi et al. [24] proposed ACFN and used spatial attention to select a subset of correlation filters for visual object tracking. On the other hand, our channel network fuses the channel coefficients from global max-pooling and global average pooling and then forwards to convolutional layer. The global max-pooling exploits the finer and distinctive target information while global average pooling reflects the overall knowledge of the target for proposed channel attention.

**Figure 2:**
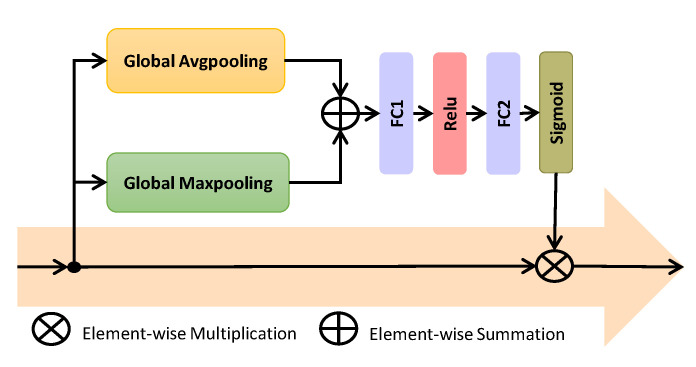
Channel attention network.

The proposed channel attention mechanism is a lightweight network, as depicted in Figure 2. The input for this network is the output features θ(z) from the last convolutional layers. This network passes the inputs to Global Average Pooling (GAP) and Global Maximum Pooling (GMP) layers. The outputs of these layers are fused using an element-wise operation to form a Global Descriptor (GD). The GD is feed forwarded to a dimensionality reduction layer, a rectifier activation layer, and a dimensionality increasing layer and then relayed to a Sigmoid activation layer to provide the final weights of the input features.

The input to the channel attentional mechanism is represented as C=θ(Z) from Equation (4). The Global Descriptor (GD) is calculated using element-wise operation (⊕) between the outputs from GAP and GMP layers as:

(5) GD=GAP(C)⊕GMP(C).

The weights for input features are computed as:

(6) $$\alpha =\sigma ({\mathrm{fc}}_{2}\left(\mathrm{Relu}\left({\mathrm{fc}}_{1}\left(GD\right)\right)\right))),$$

where ${\mathrm{fc}}_{1}$ and ${\mathrm{fc}}_{2}$ denote fully connected layers, Relu represents rectifiers layer, and σ is the Sigmoid function as $f\left(x\right)=\frac{1}{1+e^{-x}}$. It is assumed that *C* has *k* feature channels such that $C=[c_{1},c_{2},...c_{k}]$.

(7) $$\hat{c_{k}}=\alpha_{k}\times c_{k},$$

where $\alpha_{k}$ represents the $k^{th}$ weight for channel $c_{k}$. Then the final output of channel attention will be $\Delta \left(C\right)=\Delta \left(\theta \left(Z\right)\right)=\left[\hat{c_{1}},\hat{c_{2}},...\hat{c_{k}}\right]$.

The output of proposed channel attention element-wise is added to the θ(Z) using skip connection as shown in proposed framework Figure 1. Proposed channel attention is only applied in the template branch of our framework to exploit the target feature channels.

## 4. Experiments

### 4.1. Implementation Details

We train proposed model over GOT-10K dataset [53] which contains more than 10,000 video sequences. The proposed network accepts four input image patches. During offline training, the input size for the template and noisy template is 127×127×3, while that for search region and noisy search region is 255×255×3. For noisy images, μ is fixed at zero and σ is set to 0.09 which is obtained empirically and discussed in Section 4.3. During data curation, we crop the input patches such that the target object resides at the center as it reflects the most influential region for tracking performance. During training, we regularize our input using Gaussian additive noise such that it refrains to distract against noise at inference time. The model was trained offline end-to-end using a stochastic gradient method for 50 epochs. We set the momentum to 0.9 and the weight decay to $5\times 10^{-4}$, while the learning rate started at $10^{-2}$ and later decreased to $10^{-5}$. During training, we adopt the following loss function to update the model parameters:

(8) $$L\left(g,y\right)=\frac{1}{\left|\delta \right|}\sum_{(k)\in \delta }log(1+exp\left(-g\left(k\right)\times y\left(k\right)\right),$$

where *g* represents the response map, y∈{+1,−1} denotes ground-truth label, *k* shows the position in the response, and δ indicates the set of positions in the search window on the score map.

During testing, we set template and noisy template is 135×135×3, while that for search region and noisy search region is 263×263×3. During the inference, the maximum location on the response map represents the new estimated target location. To overcome the problem of scale variations, we constructed a pyramid over three scales (0.963,1,1.0375) based on previously estimated location for the current frame and selected the best score for target scale estimation. The code was implemented in python 3.7 and PyTorch 1.0.1 and all the experiments were performed using 1 GPU NVIDIA TITAN $X_{p}$ over i7 3.6GHz CPU (PRIME Z370-A II) with 32G memory.

### 4.2. Comparison with State-of-the-Art Trackers

An extensive experimental evaluation is performed for six datasets including Object Tracking Benchmark 2013 (OTB2013) [26], OTB2015 [27] TempleColor128 (TC-128) [28], UAV123 [29], VOT2016 [30], and VOT2017 [31]. OTB2013 [26] comprises 50 different challenging videos, while OTB2015 [27] is an extended version containing 100 sequences. TC-128 [28] contains 128 colored challenging sequences. UAV123 contains 123 videos captured from Unmanned Aerial Vehicle (UAV) at a low-altitude [29]. Precision and success metrics were used to perform a comparison for aforementioned datasets. The precision is computed using the Euclidean distance between the ground-truth center and the predicted center as:

(9) $$P_{gp}=\sqrt{{\left(x_{g}-x_{p}\right)}^{2}+{\left(y_{g}-y_{p}\right)}^{2}},$$

where $\left(x_{g},y_{g}\right)$ denote the ground-truth center location, and $\left(x_{p},y_{p}\right)$ represent the predicted target center position in a frame. A frame is considered successful if the precision is within a threshold of $P_{gp}$ which is set $P_{gp}$ equal to 20 pixels in the current work. Similarly, success is determined from the overlap score between the ground-truth bounding box $r_{g}$ and the predicted bounding box $r_{t}$ as:

(10) $$OS=\frac{|r_{t}\cap r_{g}|}{|r_{t}\cup r_{g}|},$$

where |·| indicate the number of pixels, ∩ shows the intersection of two regions while ∪ indicates the union of two regions. If the overlap score (OS) exceeded 0.5, the frame is classified as having been tracked successfully; otherwise, the tracking is classified as failure. We performed One Pass Evaluation (OPE) to validate our tracking method [9]. We also performed evaluation over VOT2016 [30] and VOT2017 [31]. The tracker is re-initialized during the evaluation if it encounters failure. We used the Expected Average Overlap (EAO), Accuracy (A), and Robustness (R) parameters for the evaluation for VOT2016 and VOT2017 datasets. Accuracy represents the average overlap score between estimated bounding box and ground truth. Robustness means the number of times a tracker failed. EAO computes the expected overlap score for typical short-term sequence lengths over an interval by averaging the scores for the expected average overlap curve [54].

We compared our method with 30 state-of-the-art trackers including SiamTri [55], CSRDCF [48], CNNSI [56], SRDCF [57], Staple [58], TRACA [59], SiameseFC [15], CFNet [10], ACFN [24], SiamFc-lu [60], HASiam [61], SiamFCRes22 [62], Kuai et al. [63], MSN [64], MLT [65], KCF [66], SCT [67], OA-LSTM [68], ECOhc [23], DSiam [69], MEEM [70], CCOT [40], SAMF [71], CMKCF [72], SATIN [73], GradNet [74], SiameseRPN [75] DSST [76], MemTrack [14], MemDTC [77], and UDT [78].

#### 4.2.1. Evaluation over OTB Datasets

**Figure 3:**
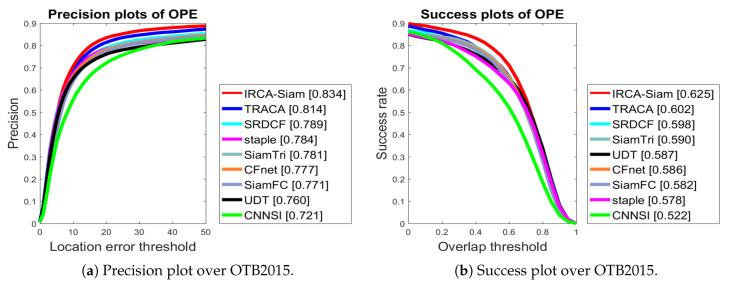
Performance comparison over OTB2015.

We present precision and success plots for the OTB2015. We compared IRCA-Siam with other state-of-the-art methods including TRACA, SRDCF, staple, SiamTri, CFNet, SiamFC, UDT, and CNNSI. Figure 3 demonstrates that the proposed algorithm IRCA-Siam showed better tracking performance compared to other trackers. IRCA-Siam achieved 62.5% and 83.5% success and precision respectively, which is 3.9% and 6.3% gain in performance compared to baseline SiamFC tracker. We compared our method with Siamese-based trackers including SiamTri, SiameseFC, CFNet, UDT, and CNNSI as shown in Figure 3. These tracking approaches take two inputs, but our approach takes four inputs. During training, we train our model such that it withholds discriminative ability for better localization. Our method has achieved 2.1% and 2.3%, 4.5% and 2.7%, and 5.3% and 4.7% superior performance in terms of precision and success, respectively, compared to correlation filter-based trackers such as TRACA, SRDCF, and Staple, respectively.

We also present the success scores for OTB2013 and OTB2015 in Table 1. The table also displays the average speed in units of Frames Per Second (FPS). The table shows that MSN [64] and HASiam [61] achieved success score more than 63.0 for OTB2013. Compared to these trackers, IRCA-Siam secured superior success score of 65.3. We also observed that our algorithm surpassed the other methods over OTB2015. Futhermore, our algorithm performs tracking at 77 FPS and is a real-time tracker. Although TRACA [59], SiamTri [55], Staple [58], SiamFC-lu [60], and SiameseFC [15] show higher tracking speed than our algorithm, they are less successful for OTB2013 and OTB2015.

#### 4.2.2. Challenge-Based Comparison

**Figure 4:**
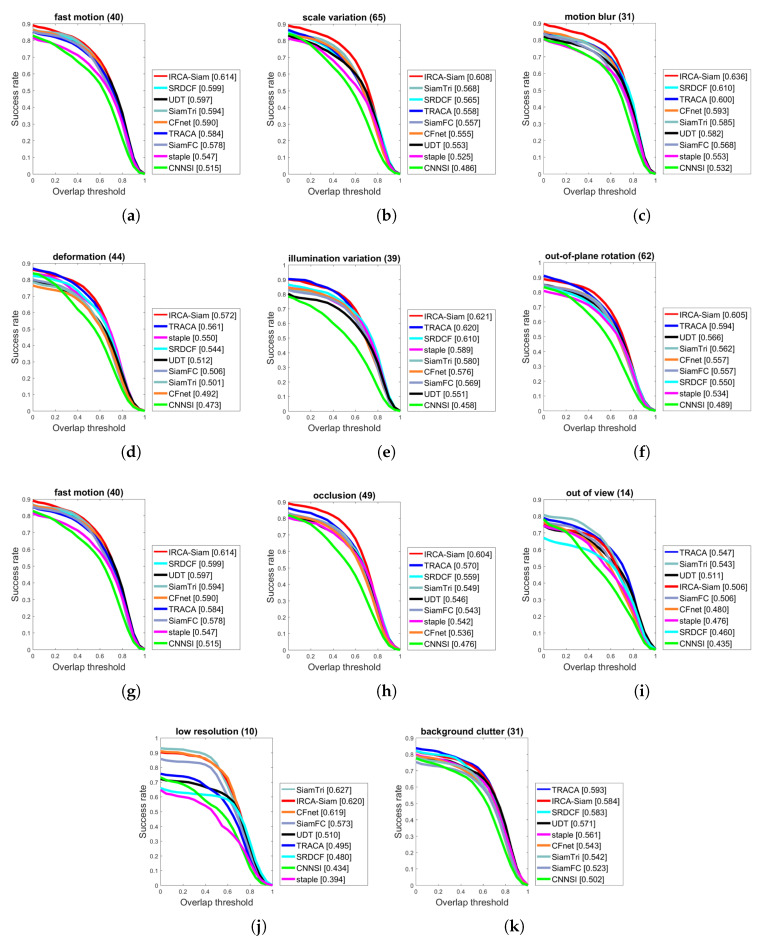
Success plots over OTB2015 for different challenges such as (**a**) fast motion, (**b**) scale variation, (**c**) motion blur, (**d**) deformation, (**e**) illumination variation, (**f**) out-of-plane rotation, (**g**) fast motion, (**h**) occlusion, (**i**) out-of-view, (**j**) low resolution, and (**k**) background clutter.

**Figure 5:**
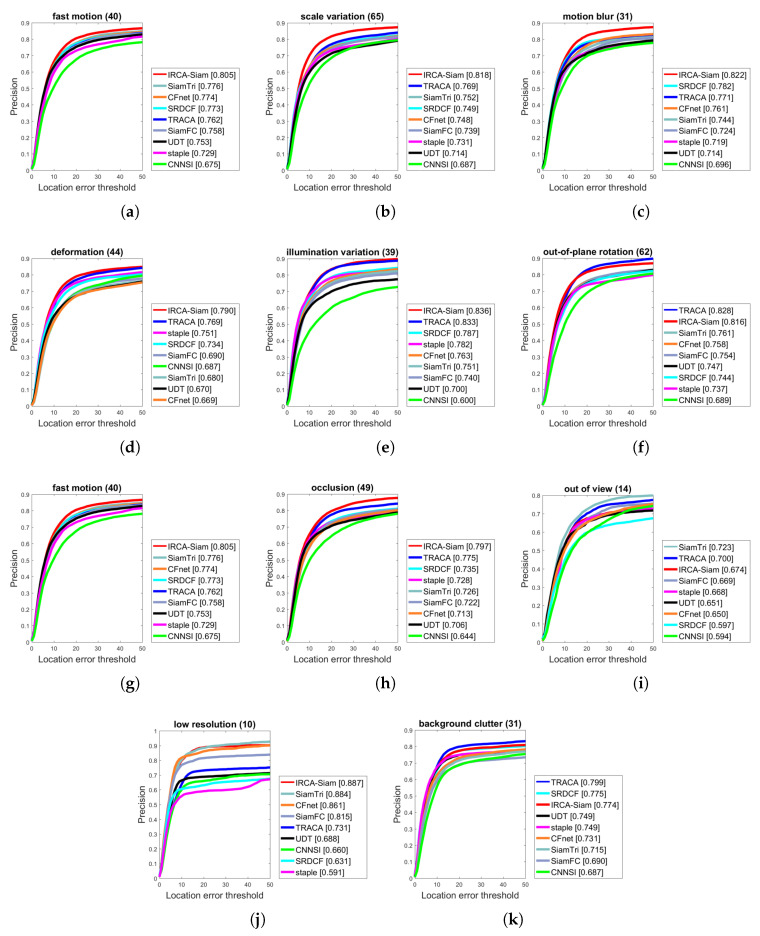
Precision plots over OTB2015 for different challenges such as (**a**) fast motion, (**b**) scale variation, (**c**) motion blur, (**d**) deformation, (**e**) illumination variation, (**f**) out-of-plane rotation, (**g**) fast motion, (**h**) occlusion, (**i**) out-of-view, (**j**) low resolution, and (**k**) background clutter.

We present the evaluation of IRCA-Siam for various tracking challenges and compared with other state-of-the-art methods including TRACA, SRDCF, staple, SiamTri, CFNet, SiamFC, UDT, and CNNSI over OTB2015 in terms of success and precision in Figure 4 and Figure 5 respectively. IRCA-Siam showed the best performance over fast motion, motion blur, deformation, in-planar rotation, out-of-planar rotation, occlusion, illumination variations, and scale variations challenges in terms of success. IRCA-Siam did not perform well over low-resolution videos and background clutter but ranked second with a minor difference as shown in Figure 4. SiamTri and TRACA surpassed our method with less than 1.0% for low-resolution and background clutter. However, overall, our tracker performed best for most of the challenges in terms of success.

We present precision plots for different challenges in Figure 5. Our algorithm showed better performance for eight challenges including fast motion, scale variations, illumination variations, occlusion, deformation, motion blur, in-plane rotation and low resolution. IRCA-Siam showed second best performance for out-of-view, low resolution, and background clutter. However, the difference between the top ranked compared with our method is less than 1.0%. As our approach ranked best for the rest of the challenges, such a minor difference can be ignored. We notice that other Siamese-based trackers are trained from raw images and do not perform well against different challenges. However, we train our model with regularized input such that it preserves the discriminative ability for better localization against noise during test time. This approach helped our method to perform better for most of the challenges in terms of both success and precision as shown in Figure 4 and Figure 5 respectively.

#### 4.2.3. Qualitative Analysis

**Figure 6:**
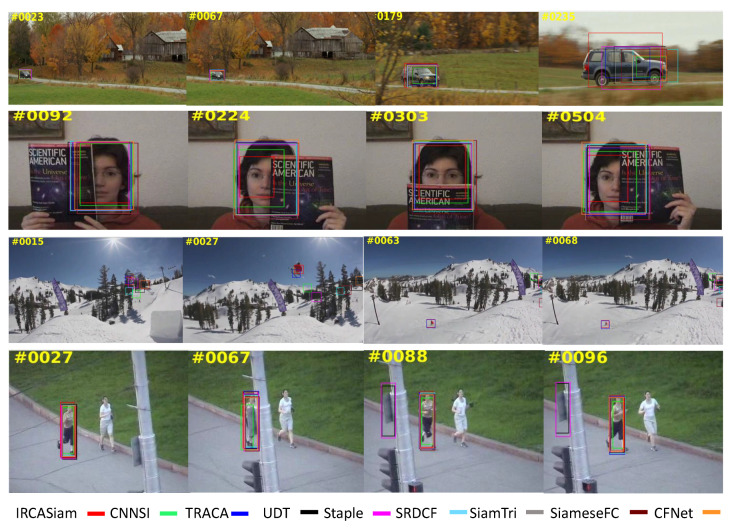
Qualitative analysis over *CarScale*, *FaceOcc1*, *Skiing*, and *Jogging-1* sequences.

We performed the qualitative analysis of the proposed method over *CarScale*, *FaceOcc1*, *Skiing*, and *Jogging-1* sequences as shown in Figure 6. In *CarScale* sequence, IRCA-Siam performed better compared to others as its bounding box enclose most region of the vehicle while others less. Almost all the trackers tackled the *FaceOcc1* sequence successfully. However, IRCA-Siam and TRACA succeeded to track the skier in *Skiing* sequence. The proposed method also performed efficiently for occlusion in *Jogging-1* sequence.

#### 4.2.4. Evaluation over TC128 Dataset

We validate the proposed IRCA-Siam tracker over TC128 benchmark dataset and showed the precision and success in Table 2. We compared our method with UDT [78], Kuai et al. [63], KC [66], MLT [65], SCT [67], SiameseFC [15], CFNet [10], Staple [58], CNNSI [56], OA-LSTM [68], and SRDCF [57]. The proposed method secured the first rank compared to other trackers with maximum precision score 74.5 and success 55.0.

#### 4.2.5. Evaluation over UAV123 Dataset

This benchmark contains 123 videos captured from an Unmanned Aerial Vehicle (UAV) at a low-altitude. We opted to validate the proposed method over UAV123 dataset and showed the precision and success in Table 3. We compared IRCA-Siam with trackers including MLT [65], Kuai et al. [63], KCF [66], SRDCF [57], ECOhc [23], MEEM [70], SAMF [71], and DSST [76]. The results showed that IRCA-Siam demonstrated outstanding performance compared to other methods and secured best precision 74.5 and success 52.0.

#### 4.2.6. Evaluation over VOT2016 and VOT2017 Dataset

We present the performance comparison over VOT2016 and VOT2017 in Table 4 and Table 5 respectively. We compared our method over VOT2016 with various state-of-the-art trackers such as MemTrack [14], MemDTC [77], ECO [23], HASiam [61], Staple [58], SRDCF [57], DSiam [69], MLT [65], CCOT [40], UDT [78], SiameseFC [15], CMKCF [72], and SiamFCRes22 [62]. We observe that CCOT [40] secured best EAO 0.33 but our IRCA-Siam algorithm showed better accuracy and robustness for VOT2016 dataset. CMKCF [72] have shown lower robustness compared to our method but its accuracy is lower than ours. Moreover, our method showed best accuracy 0.56 compared to other state-of-the-art methods for VOT2016.

Performance comparison over VOT2017 is shown in Table 5. We compared our tracker with other trackers over VOT2017 dataset are CSRDCF [48], MemTrack [14], MemDTC [77], SRDCF [57], MSN [64], DSST [76], SATIN [73], SiameseFC [15], GradNet [74], SiameseRPN [75], and SiamFCRes22 [62]. We note that SATIN [73] showed best EAO score but its accuracy and robustness it not better than our algorithm. Furthermore, our algorithm showed best accuracy 0.52 and robustness 0.29 compared to other state-of-the-art algorithms.

### 4.3. Ablation Study

**Figure 7:**
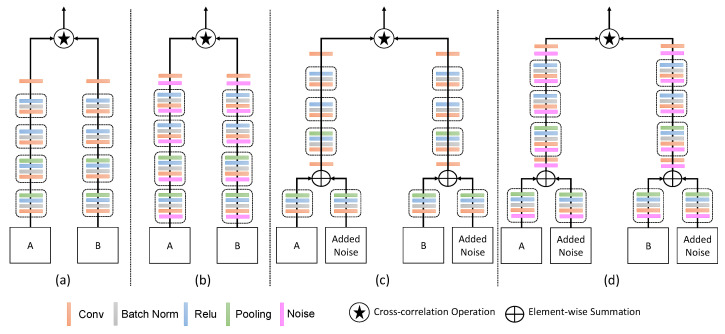
Added noise to inputs and different convolutional layers for SiameseFC and proposed framework. (**a**) shows the baseline SiameseFC, (**b**) indicates the SiameseFC with noise layers before convolutional layers, (**c**) represents the proposed framework without channel attention, and (**d**) shows the proposed framework with noise layers before convolutional layers and without channel attention.

In this section, we investigate the effect of input additive noise and noise layers before convolution layers during the training. During testing, we neither provide input noise nor noise layers. We performed different experiments for SiameseFC and proposed (IR-Siam) method as shown in Figure 7. We also evaluated the performance of the proposed channel attention with additive noise named IRCA-Siam as shown in Figure 1. We performed our ablation study over OTB2015 dataset and showed the performance in precision and success.

In our framework, noise is added to inputs as regularization instead of dropout approach. Liu et al. [36] used noise layer to prevent their network from adversarial attacks. Therefore, we also used noise layer before convolutional layers to verify the improvement of generalization error using noise layer within convolutional model θ. We present the additive noise as input regularization as well as noise layer within Siamese tracking framework as shown in Figure 7. In Figure 7a shows the baseline SiameseFC tracking framework. We used a noise layer and placed before each convolutional layer to learn the noisy gradients during back propagation. Figure 7b presents the SiameseFC with noise layer before each convolutional layer. Similarly, Figure 7c,d represents the proposed framework without channel attention and, with and without noise layer, respectively. In our ablation study, we preformed different experiments to show the impact of addition of noise layer within Siamese framework.

**Figure 8:**
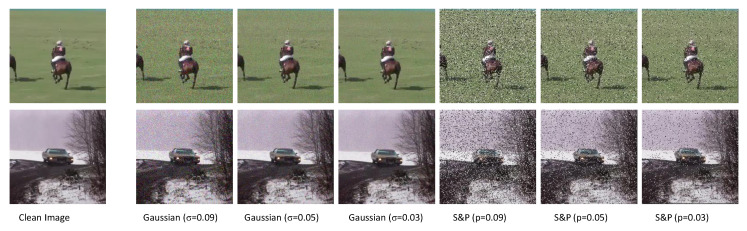
Illustration of additive noises to inputs. Here σ represents the variance of Gaussian noise while p denotes the probability for Salt and Pepper (S&P) noise.

First, we evaluate the performance of additive input noise. In this study, we used Salt and Pepper (S&P) and Gaussian noise as input noise. For S&P noise, we use three different probabilities (0.09, 0.05, and 0.03), similarly we use three different σ (0.09, 0.05, and 0.03) with mean (μ) zero for Gaussian input noise computation as shown in Figure 8. We observe that SiameseFC showed better performance without addition of noise. On the other hand, our IR-Siam without channel attention improved the tracking performance in the addition of Gaussian noise with σ = 0.09 and achieved precision = 81.9 and success = 61.9.

We investigate the addition of input layers within the network architecture. We only added Gaussian noise layers before convolution layers as shown in Figure 7. We observe that the added noise layer degrades the performance for SiameseFC as well as our IR-Siam tracker. From Table 6, we note that IR-Siam shows the tracking improvement when noise is added as input. Moreover, we also find that the added channel attentional module shows tracking performance improvement. Proposed IRCA-Siam with channel attention achieved best precision = 83.4 and success = 62.5. The improved performance of IRCA-Siam reflects the importance of proposed channel attention network as it efficiently highlights the important feature channels and reduces the significance of the irrelevant ones.

## 5. Conclusions

In this work, input-noise-based regularization is proposed to improve tracking generalization. In addition, early feature fusion of noisy and clean channels is also proposed for better target localization. In the same framework, channel attention has been proposed to select more informative target features to improve tracking performance. For input-noise regularization, Gaussian noise has been added to both the template and the search patches during the training. Feature fusion is performed at low-level layers to make the tracking process more robust to noise and to improve target localization. Channel attention has been used to highlight more descriptive features and to suppress the noisy features. The proposed tracker has shown superior performance compared to 18 Siamese trackers and 12 other existing trackers. The proposed tracker has shown promising performance for fast motion, motion blur, deformation, in-plane rotation, out-of-plane rotation, occlusion, illumination variations, and scale variation challenges.

## Figures and Tables

**Table 1:** Performance comparison of IRCA-Siam with other trackers over OTB2013 and OTB2015 using success and speed in FPS.

Tracker	OTB2013	OTB2015	FPS	Real-Time
TRACA [59]	65.2	60.3	**101**	Yes
SiamTri [55]	61.5	59.0	85	Yes
CSRDCF [48]	59.9	58.2	24	No
ACFN [24]	60.7	57.5	15	No
CNNSI [56]	53.9	52.2	<1	No
SRDCF [57]	62.6	59.8	6	No
Staple [58]	59.3	57.8	80	Yes
SiamFc-lu [60]	-	62.0	82	Yes
HASiam [61]	64.0	61.1	30	Yes
Kuai et al. [63]	-	62.2	25	No
MSN [64]	64.3	59.7	40	Yes
MLT [65]	62.1	61.1	48	Yes
SiameseFC [15]	60.7	58.2	86	Yes
CFNet [10]	58.9	58.6	43	Yes
UDT [78]	61.9	58.7	70	Yes
**IRCA-Siam**	**65.3**	**62.5**	77	Yes

**Table 2:** Comparison of the proposed method with various state-of-the-art methods over TC128 using precision, success and speed in FPS.

Trackers	Precision	Success	FPS
UDT [78]	71.7	50.7	70
Kuai et al. [63]	71.6	52.3	25
KCF [66]	54.9	38.7	160
MLT [65]	-	49.8	48
SCT [67]	62.7	46.6	40
SiameseFC [15]	68.8	50.3	86
CFNet [10]	60.7	45.6	43
Staple [58]		49.8	80
CNNSI [56]	63.8	44.8	<1
OA-LSTM [68]	70.8	49.5	11.5
SRDCF [57]	-	50.9	6
**IRCA-Siam**	74.5	55.0	77

**Table 3:** Comparison of the proposed method with various state-of-the-art methods over UAV123 using precision and success.

Trackers	Precision	Success
MLT [65]	-	43.5
Kuai et al. [63]	73.0	50.9
KCF [66]	54.9	38.7
SRDCF [57]	67.7	46.4
ECOhc [23]	72.5	50.6
MEEM [70]	62.7	39.2
SAMF [71]	59.2	39.6
DSST [76]	58.6	35.6
**IRCA-Siam**	74.5	52.0

**Table 4:** Performance comparison for different trackers over VOT2016.

Trackers	Overlap (↑)	Robustness (↓)	EAO (↑)
MemTrack [14]	0.53	1.44	0.27
MemDTC [77]	0.51	1.82	0.27
ECO [23]	0.54	-	0.37
HASiam [61]	-	-	0.27
Staple [58]	0.53	0.38	0.29
SRDCF [57]	0.54	0.42	0.25
DSiam [69]	0.49	2.93	0.18
MLT [65]	0.53	-	-
CCOT [40]	0.54	0.24	**0.33**
UDT [78]	0.54	-	0.22
SiameseFC [15]	0.53	0.46	0.23
CMKCF [72]	0.53	**0.18**	0.30
SiamFCRes22 [62]	0.54	0.38	0.30
**IRCA-Siam**	**0.56**	0.19	0.30

**Table 5:** Performance comparison for different trackers over VOT2017.

Trackers	Overlap (↑)	Robustness (↓)	EAO (↑)	FPS
CSRDCF [48]	0.49	0.49	0.25	13
MemTrack [14]	0.49	1.77	0.24	50
MemDTC [77]	0.49	1.77	0.25	40
SRDCF [57]	0.49	0.97	0.12	6
MSN [64]	0.50	0.46	0.26	40
DSST [76]	0.39	1.45	0.08	24
SATIN [73]	0.49	1.34	**0.28**	24
SiameseFC [15]	0.50	0.59	0.19	86
GradNet [74]	0.50	0.37	0.24	80
SiameseRPN [75]	0.49	0.46	0.24	200
SiamFCRes22 [62]	0.50	0.49	0.23	70
**IRCA-Siam**	**0.52**	**0.29**	0.25	76

**Table 6:** Ablation study performed over OTB2015 using precision and success.

Tracker	Additive Input Noise	Added Noise Layer before	Added Noise Layer Type	Precision	Success
SiameseFC	-	-	-	77.1	58.2
SiameseFC	S&P (p = 0.09)	-	-	76.5	57.2
SiameseFC	S&P (p = 0.05)	-	-	75.2	54.8
SiameseFC	S&P (p = 0.03)	-	-	73.5	52.9
SiameseFC	Gaussian (μ=0,σ=0.09)		-	76.9	57.8
SiameseFC	Gaussian (μ=0,σ=0.05)	-	-	75.7	56.4
SiameseFC	Gaussian (μ=0,σ=0.03)	-	-	75.1	55.3
SiameseFC	-	Conv5	Gaussian (μ=0,σ=0.09)	76.8	56.5
SiameseFC	-	Conv5	Gaussian (μ=0,σ=0.05)	75.2	55.7
SiameseFC	-	Conv5	Gaussian (μ=0,σ=0.3)	74.1	53.9
SiameseFC	-	Conv1, Conv2, Conv3, Conv4, Conv5	Gaussian (μ=0,σ=0.09)	75.5	55.9
SiameseFC	Gaussian (μ=0,σ=0.09)	Conv1, Conv2, Conv3, Conv4, Conv5	Gaussian (μ=0,σ=0.09)	76.7	57.9
IR-Siam	-	-	-	80.8	60..6
IR-Siam	S&P (p = 0.09)	-	-	81.6	61.5
IR-Siam	S&P (p = 0.05)	-	-	80.3	61.0
IR-Siam	S&P (p = 0.03)	-	-	79.9	59.3
IR-Siam	Gaussian (μ=0,σ=0.09)	-	-	81.9	61.9
IR-Siam	Gaussian (μ=0,σ=0.05)	-	-	81.2	61.3
IR-Siam	Gaussian (μ=0,σ=0.03)	-	-	80.1	60.4
IR-Siam	-	Conv6	Gaussian (μ=0,σ=0.09)	80.9	60.6
IR-Siam	-	Conv6	Gaussian (μ=0,σ=0.05)	80.2	60.1
IR-Siam	-	Conv6	Gaussian (μ=0,σ=0.03)	78.9	58.7
IR-Siam	-	Conv1, Conv2, Conv3, Conv4, Conv5, Conv6	Gaussian (μ=0,σ=0.9)	81.2	60.7
IR-Siam	Gaussian (μ=0,σ=0.09)	Conv1, Conv2, Conv3, Conv4, Conv5, Conv6	Gaussian (μ=0,σ=0.09)	80.5	59.5
IR-Siam	-	Conv1, Conv2, Conv6	Gaussian (μ=0,σ=0.09)	81.5	60.5
IR-Siam	Gaussian (μ=0,σ=0.09)	Conv1, Conv2, Conv6	Gaussian (μ=0,σ=0.09)	82.1	60.8
IRCA-Siam	S&P (p = 0.09)	-	-	82.7	62.3
IRCA-Siam	Gaussian (μ=0,σ=0.09)	-	-	83.4	62.5
